# Differential Gene Expression Profiling and Biological Process Analysis in Proximal Nerve Segments after Sciatic Nerve Transection

**DOI:** 10.1371/journal.pone.0057000

**Published:** 2013-02-21

**Authors:** Shiying Li, Qianqian Liu, Yongjun Wang, Yun Gu, Dong Liu, Chunming Wang, Guohui Ding, Jianping Chen, Jie Liu, Xiaosong Gu

**Affiliations:** 1 Jiangsu Key Laboratory of Neuroregeneration, Nantong University, Nantong, China; 2 School of computer science and technology, Nantong University, Nantong, China; 3 Key Lab of Systems Biology, Shanghai Institutes for Biological Sciences, Chinese Academy of Sciences, Shanghai, China; Rutgers University, United States of America

## Abstract

After traumatic injury, peripheral nerves can spontaneously regenerate through highly sophisticated and dynamic processes that are regulated by multiple cellular elements and molecular factors. Despite evidence of morphological changes and of expression changes of a few regulatory genes, global knowledge of gene expression changes and related biological processes during peripheral nerve injury and regeneration is still lacking. Here we aimed to profile global mRNA expression changes in proximal nerve segments of adult rats after sciatic nerve transection. According to DNA microarray analysis, the huge number of genes was differentially expressed at different time points (0.5 h–14 d) post nerve transection, exhibiting multiple distinct temporal expression patterns. The expression changes of several genes were further validated by quantitative real-time RT-PCR analysis. The gene ontology enrichment analysis was performed to decipher the biological processes involving the differentially expressed genes. Collectively, our results highlighted the dynamic change of the important biological processes and the time-dependent expression of key regulatory genes after peripheral nerve injury. Interestingly, we, for the first time, reported the presence of olfactory receptors in sciatic nerves. Hopefully, this study may provide a useful platform for deeply studying peripheral nerve injury and regeneration from a molecular-level perspective.

## Introduction

Peripheral nerve injuries are quite common in clinical practice. Fortunately, the injured peripheral nerves have an ability to regenerate by their own. Clinicians caring for patients with peripheral nerve injury must possess a clear understanding of the response of peripheral nervous system (PNS) to trauma. During peripheral nerve regeneration, successful neuronal survival and target reinnervation depend on the intrinsic regenerative capacity of peripheral axons and the existence of a growth-permissive microenvironment [1]–[5]. Following axonal transection, a series of pathologic events occurs in the neuronal cell body and axons, both of which are interdependent in recovery [6]. Previous study reported that transection of the peripheral axons resulted in profound alterations in their metabolism, regenerative capacity, survival, excitability, transmitter function and sensitivity to diverse extrinsic and intrinsic signals [7], [8]. In addition,the injured peripheral nerves displayed stereotypic histopathological responses, indicating activation of a coordinated gene expression program [9].

The process of peripheral nerve injury and regeneration has been studied mostly from the morphological viewpoint [3]–[6]. Perhaps it is more important, however, to understand the molecular basis of the morphological change and local microenvironment regulation by analyzing the gene expression changes during peripheral nerve injury and regeneration. This is because screening and development of new treatment strategies are usually achieved through targeting the regulation of key genes. Therefore, many studies have identified that a number of genes are up-regulated or down-regulated after peripheral nerve injury [9], [10]. Global knowledge of gene expression changes and the related biological processes during peripheral nerve injury and regeneration, however, is still lacking. To further improve therapeutic and preventative approaches to peripheral nerve injury, we need to have an in-depth insight into the molecular mechanisms regulating peripheral nerve regeneration from much broader perspectives.

Microarray techniques provide the capacity to analyze parallel changes in many thousands of genes, and have been used successfully to monitor gene expression profile changes in many neural and nonneural systems [8]–[11]. DNA microarray-based expression profiling allows identification of the spectrum of genes expressed during peripheral nerve injury and regeneration. The goals of this study were to obtain global knowledge of gene expression changes following peripheral nerve injury and examine the related biological processes. DNA microarray technology was applied to monitor the mRNA expressions in proximal nerves at 0.5, 1, 3, 6, and 9 h, and 1, 4, 7, and 14 d after sciatic nerve transection in adult rats. Then, we used gene ontology (GO) enrichment analysis to determine which biological processes were related to the differential gene expressions.

## Results and Discussion

### Gene Expression Profiling in the Proximal Sciatic Nerve Segments

DNA microarray analysis was performed with the RNA sample extracted from the proximal segments of sciatic nerves at different time points post nerve transection. All the biological replicates (triple repeats for each time point) shared significant homology in the gene expression. The number of genes with the time-dependent differential expression increased after nerve injury, reaching a peak value at 7 d post nerve transection. Specifically, the number of upregulated genes was significantly larger than that of downregulated genes before 9 h post nerve transection, indicating that the stress response immediately after nerve transection was accompanied with activation of many genes. During 1–7 d post nerve transection, the number of both upregulated and downregulated genes was maintained at a large value, and the number of upregulated genes was still significantly larger than that of downregulated genes. At 14 d post nerve transection, the number of downregulated genes was hardly changed, while the number of upregulated genes decreased to be almost the same value as that of downregulated genes (Table 1).

**Table 1:** Total genes differentially expressed in proximal nerve segments at different time points post nerve transaction.

Fold change		0.5 h	1 h	3 h	6 h	9 h	1 d	4 d	7 d	14 d
2–10	Up	251	325	385	617	880	2194	1845	2114	1217
	Down	75	94	179	221	443	964	1309	1294	1127
10–50	Up	9	10	38	55	74	90	73	96	58
	Down	1	3	0	1	3	4	29	15	14
>50	Up	1	3	7	13	12	8	13	7	1
	Down	0	2	0	0	1	0	3	1	1
Total	Up	261	338	430	685	966	2292	1931	2217	1276
	Down	76	99	179	222	447	968	1341	1310	1142

The expression fold change in these genes can be classified into 3 types: the fold change of 2−10, 10–50, and >50. For the first type, the number of up-regulated genes dramatically increased during 9 h to 1 d post nerve transection, reaching the maximum at 1 d post nerve transaction, and then leveled off until 7 d post nerve transection, while the number of down-regulated genes dramatically increased, reached the maximum at 4 d post nerve transection, and leveled off until 14 d post nerve transection. For the second type, the number of up-regulated genes increased during 1 to 3 h post nerve transection, and leveled off during 9 h to 7 d post nerve transection, while the number of down-regulated genes increased and reached the maximum at 4 d post nerve transection, followed by an ensuing decrease. For the third type, the number of up-regulation genes increased constantly with the maximum value at 6 h, 9 h or 4 d post nerve transaction (Table 1). These changes suggested that the gene upregulation might occur before gene down-regulation, and that nerve transection might stimulate the up-regulation of a few genes, which initiated the expression of more genes in a hierarchical manner.

### Confirmation of Differential Gene Expression of Several Genes

**Figure 1:**
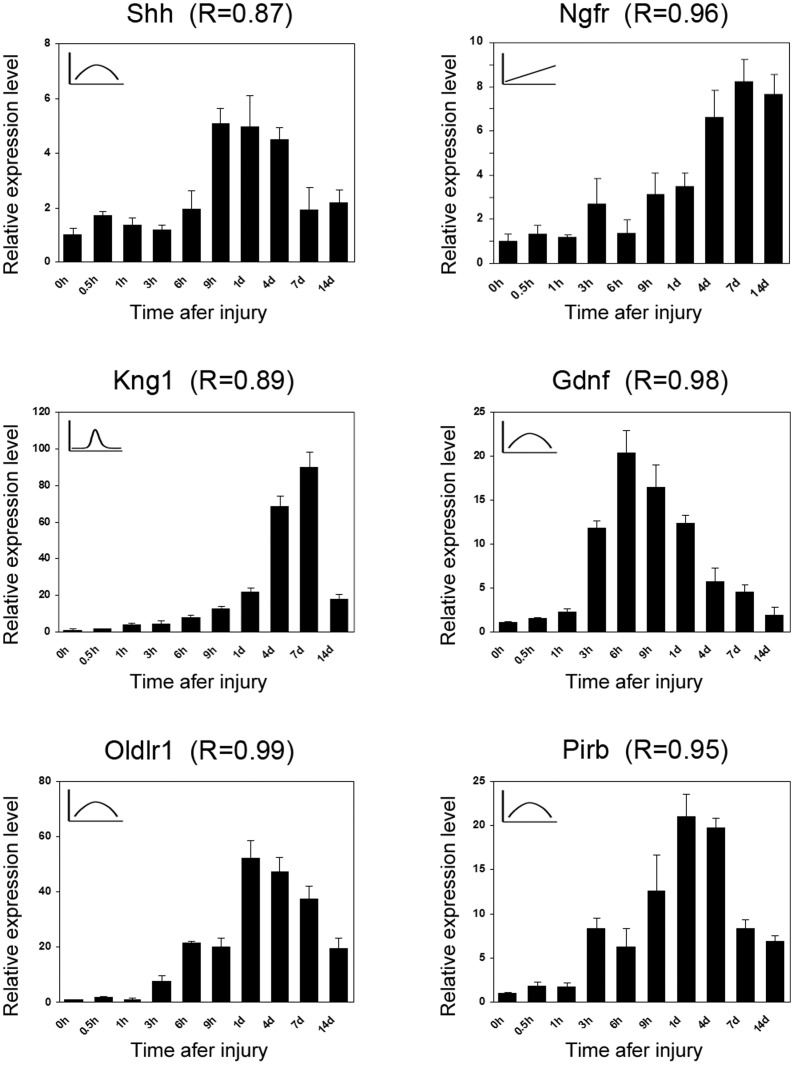
Confirmation of several gene expressions by real-time RT-PCR. Relative mRNA levels of Ngfr, Shh, Gdnf, Pirb, Oldlr1 and kng1 were analyzed by real-time RT-PCR at different time points post nerve transection (control: 0 h). The expression profile determined by microarray analysis is schematically shown in the upper left corner of each pane. Data are presented as means ± SD of three dependent assays (each in triplicate). R stands for the correlation coefficient.

In this study, microarray data showed a good repeatability between 3 biological replicates at each time point, and the microarray data for some genes, for example, Ngfr, Shh, and IL-1, are in accordance with the previously reported results [12]–[14], indicating the reliability of our microarray analysis. In order to verify the microarray data, however, 6 differentially expressed genes (Ngfr, Shh, Gdnf, Pirb, Oldlr1, and Kng1) were randomly selected and then subjected to real-time quantitative RT-PCR analysis. The results indicated the expression of such 6 genes showed the change trends similar to those revealed by microarray data, and the correlation analysis suggested that qRT-PCR data were strongly correlated to microarray data (Figure 1).

### Biological Process Analysis

**Figure 2:**
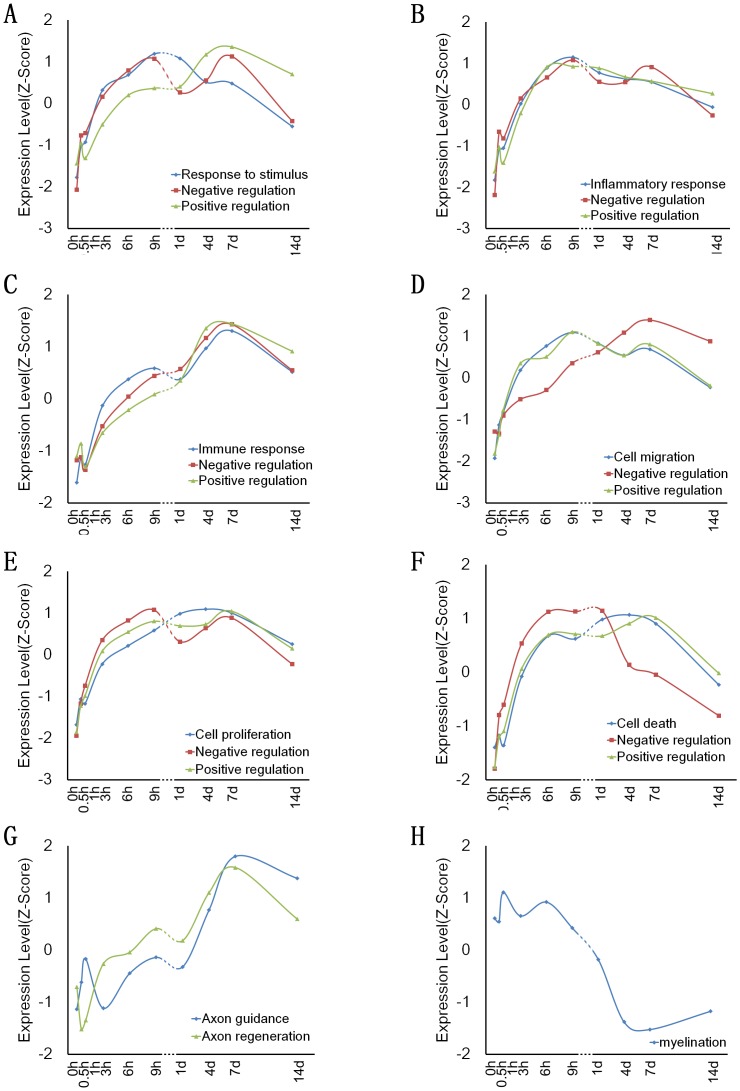
The average expression pattern and change trend of major biological processes involving differentially expressed genes in the proximal sciatic nerve sample at different time points post nerve transection.

**Figure 3:**
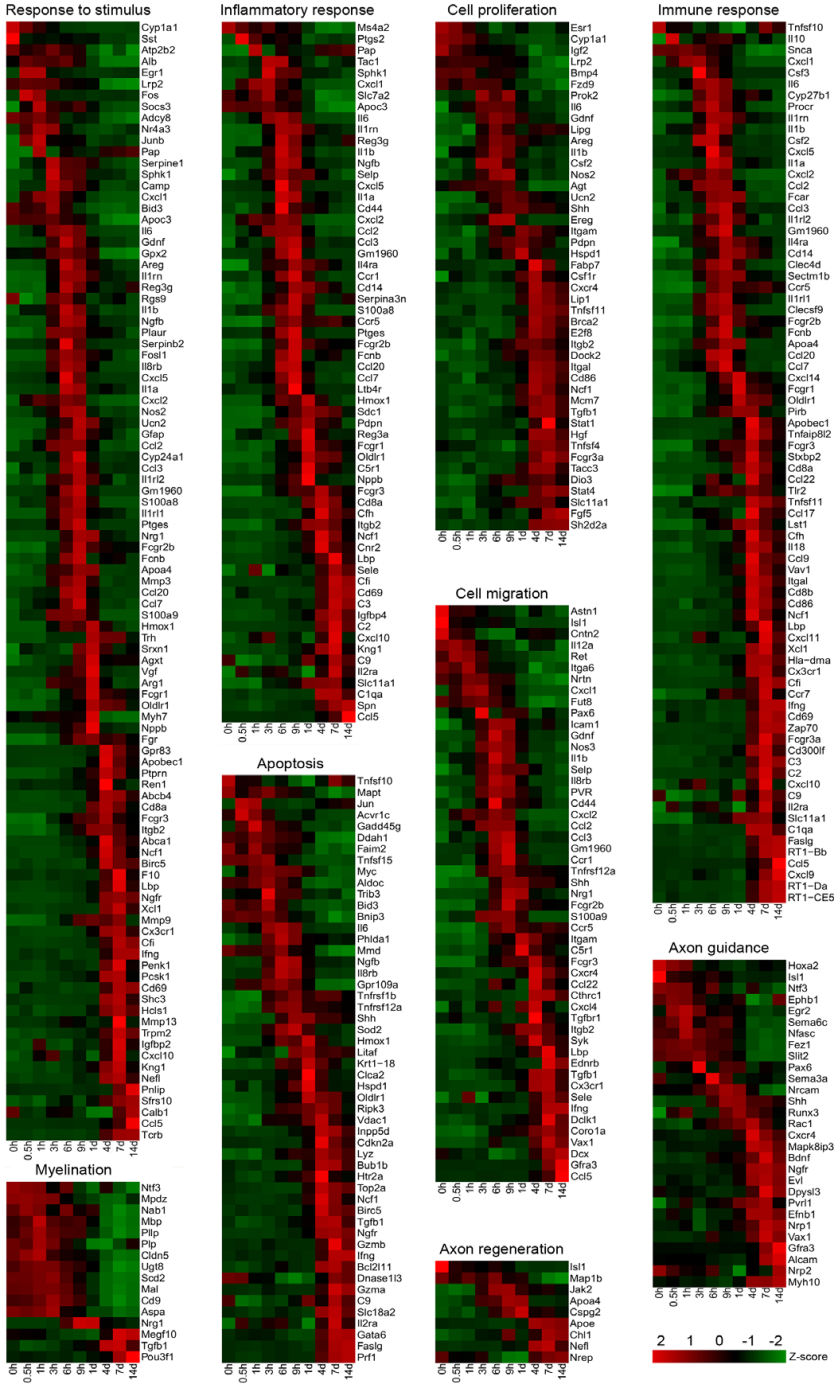
Hierarchical clustering of key regulatory genes involved in designated biological processes during sciatic nerve regeneration.

The biological processes involving differential gene expressions were identified by GO enrichment analysis using the DAVID online tool. Our attention was focused on 9 classes of biological processes, i.e. detection of stimulus, response to stimulus, inflammatory response, immune response, cell proliferation, cell migration, cell death, axon regeneration and guidance, and myelination. Most of these biological processes maintained the relative balance through negative or positive regulation, which was also our concern. Genes were grouped together according to their involvement in the same biological process. Several genes might be involved in more than one biological process, and each of them may belong to multiple groups. The number of differentially expressed genes in various biological processes at each time point is showed in Table 2. For 7 classes of biological processes, the number of genes was a maximum value at 7 d post nerve transection, but for 2 classes of biological processes, (detection of stimulus and cell death), the number of genes was a maximum value at 1 and 4 d post nerve transection, respectively. On the other hand, the gene number under negative or positive regulation was a maximum value at 7 or 4 d post nerve transection (Table 2). In order to analyze the change trend of biological process involving differentially expressed genes, the average expression profiles of differentially expressed genes in each of the biological processes (except detection of stimulus) were calculated and shown in Figure 2, and the corresponding differentially expressed genes were listed in Table S1.1−S1.8. In addition, according to the fold change of differentially expressed genes and by referring to the literature, we selected key regulatory genes for hierarchical clustering (Figure 3, Figure S1). The expression of these selected genes at different time points post nerve transaction were listed in Table S2.1−S2.8, the fold change of these genes relative to control (0 h) was shown in Table S3.1−S3.8, and the expression profiles of these genes were shown in Table S4. The brief descriptions for the 9 classes of biological processes are provided as follows:

**Table 2:** Total genes that were differentially expressed and involved in 9 classes of biological processes at different time points post nerve transaction.

Biological process	0.5 h	1 h	3 h	6 h	9 h	1 d	4 d	7 d	14 d
**Detection of stimulus**	19	74	25	17	104	442	123	298	139
**Response to stimulus**									
response to stimulus	51	64	120	187	220	350	413	449	308
Positive regulation	9	12	15	29	41	60	83	82	66
Negative regulation	6	–	9	14	6	11	35	32	24
**Inflammatory response**									
Inflammatory response	15	19	34	62	63	76	90	93	72
Positive regulation	–	–	5	13	15	12	17	12	13
Negative regulation	–	–	5	9	8	6	10	10	8
**Immune response**									
Immune response	20	20	52	72	92	112	135	173	120
Positive regulation	6	10	9	19	27	44	57	57	48
Negative regulation	–	–	–	–	–	–	14	16	11
**Cell migration**									
Migration	9	13	29	36	45	61	65	75	56
Positive regulation	–	3	9	13	16	13	13	29	16
Negative regulation	–	4	3	5	7	7	15	11	10
**Cell proliferation**									
Proliferation	8	5	21	35	42	60	63	71	47
Positive regulation	10	–	28	39	53	83	95	97	71
Negative regulation	10	–	22	34	43	58	65	72	56
**Cell death**									
Death	9	13	24	36	51	49	85	84	68
Positive regulation	12	10	28	36	46	43	86	79	62
Negative regulation	14	12	29	39	46	77	92	76	54
**Axon**									
Axon guidance	–	5	6	4	11	10	22	27	23
Axon regeneration	–	–	–	–	6	4	–	5	5
**Myelination**	–	–	–	–	–	3	15	18	14

#### 1) Detection of stimulus

**Figure 4:**
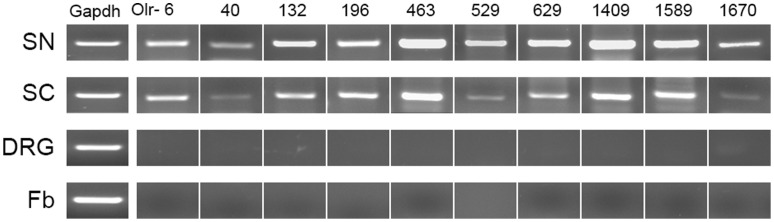
Validation of olfactory receptor expression in sciatic nerves. A representative semi-quantitative RT-PCR image showing the expression of 10 members of olfactory receptors in the sciatic nerve (SN), Schwann cell (SC), L4–6 DRG neuron (DRG) and fibroblast (Fb) samples.

Detection of stimulus is a sequences of events in which a stimulus is received by a cell or organism and then converted into a molecular signal. Following sciatic nerve transection, proximal nerves will detect stimulus signal for triggering responses. In this study, we first observed the class of biological process called “detection of stimulus”, and determined time-dependent differential expression of the related genes. It was surprising that most of this bioprocess involved olfactory receptors (Table S1.9). Especially, the expression of many olfactory receptors, such as Olfr-40, 463, 629, 728, 1108 and 1589, was changed at most of time points examined. In order to verify microarray data, 10 olfactory receptors were randomly selected for confirmation of their expression by RT-PCR. The clear band of olfactory receptors was observed with the correct molecular weight (Figure 4), suggesting that olfactory receptors might be present in sciatic nerves. To further determine in which cell types olfactory receptors were expressed, we applied RT-PCR to the main cell types contained in sciatic nerves, including Schwann cells, fibroblasts, and DRG (L4–6) neurons. The resulting data suggested that the olfactory receptors were likely to be mainly expressed in Schwann cells (Figure 4). This finding was supported by an observation that the expression differences among 10 olfactory receptors in sciatic nerves were similar to those in Schwann cells (Figure 4).

As is known, olfactory receptors expressed in the cell membrane of olfactory receptor neurons are responsible for detectioning odor molecules [15], [16]. However, some tissues and cells, such as testis [17], prostate [18], notochord [19], heart [20], taste tissue [21] and primordial germ cells [22], also expressed some olfactory receptors with unclear function. This study, for the first time, showed that a large number of olfactory receptors were expressed in Schwann cells of sciatic nerves. Additionally, the expression of olfactory receptors in sciatic nerve was changed post nerve transection, implying that olfactory receptors might exert some functions other than odor detection.

#### 2) Response to stimulu

After detection of the stimulus signal induced by sciatic nerve transection, proximal nerves experience will trigger the class of biological process called “responses to stimulus”. In this study, we observed that the expression of genes involved in response to stimulus was rapidly up-regulated following sciatic nerve transection, reaching the maximum at 9 h post nerve transection, and then slowly down-regulated. Correspondingly, the expression of genes that regulates response to stimulus, including negative regulation and positive regulation, was also increased quickly at early phase. Specifically, the expression of genes involved in negative regulation showed more prompt increase than that involved in positive regulation before 9 h post nerve transection. However, the expression of genes involved in negative regulation has a fall after 9 h post nerve transection, and the expression of genes in positive regulation slowly increased until 7 d post nerve transection (Figure 2A). In addition, the genes involved in response to stimulus owned the largest fold change at 9 h post nerve transection (Table S3.1), but a large number of differentially expressed genes appeared at 7 d post nerve transection (Figure 3, Table S3.1).

Following sciatic nerve transection, the injury site will receive various extrinsic and intrinsic stimulus signals and trigger responses to stimulus. The early responses are mainly those to wounding, external stimulus and oxidative stress. After these responses, the sciatic nerve will begin to regenerate owing to activation of responses to endogenous stimulus. This might account for the temporary fall of the negative regulation for response to stimulus at 9 h after transection.

#### 3) Inflammatory response

Injury stimuli usually lead to inflammatory responses, a protective attempt to remove the injury stimuli. These inflammatory responses involve multiple cell types and effector molecules with both positive and negative effects [23]. Inflammation is essential for normal regeneration in the peripheral nervous system, nerve injury invariably elicits inflammation [24]. In this study, we observed that the expression of genes involved in inflammatory response was upregulated sharply after nerve transection and reached the maximum at 9 h post nerve transection. The expression of genes involved in positive regulation of inflammatory responses was also up-regulated sharply and reached the maximum at 6 h post nerve transection. In order to maintain the balance of inflammatory responses, the expression of genes involved in negative regulation of inflammatory responses was synchronously upregulated before 9 h post nerve transaction and then downregulated but with a temporary rebound at 7 d post nerve transection (Figure 2B). The largest fold change and the maximum number of genes involved in inflammatory response appeared 6–9 h post nerve transaction (Table S3.2). A rather high fold change appeared in proinflammatory cytokines, such as Il6, Il1α, Il1β, Il1rn (Figure 3, Table S3.2). In addition, chemokines, *e.g.*, Cxcl1, Ccl2, Ccl20, Ccl3, Cxcl2, Ccl7 Cxcl5, Ccr1 and Ccr5, were obviously upregulated at 6–9 h post nerve transection (Figure 3, Table S3.2).

#### 4) Immune response

The immune system protects the body from potential internal or invasive threat. The interactions between the immune system and the nervous system are extensive and complex [23]. Innate immune cells, T and B lymphocytes play important role in regulating axon regeneration, macrophage recruitment and myelin clearance [23], . In this study, our data revealed that either the expression of genes involved in immune responses or negative and positive regulation of immune responses were significantly increased after nerve transaction with a bit fluctuation at 1 h post nerve transection, and reach the maximum at 7 d post nerve transection (Figure 2C).

Inflammation is one of the first responses of the immune system to infection [27], and immune and inflammatory responses involve many overlapping genes, especially at initial phase. With the passage of time, the byproduct of inflammation responses and cell debris are to be cleared, leading to the continuous strengthening of immune responses. In this study, we observed that the expression of macrophage cell active genes, such as Lbp, Fcgr3a,Cx3cr1, and the genes involved in antigen processing presenting, such as Cd8a, Cd8b, Tnfsf11, Itgal were upregulated at 4 d and 7 d post nerve transection (Figure 3, Table S3.3).

#### 5) Cell migration

Cell migration is a central event in the development and maintenance of multicellular organisms. Tissue formation during wound healing and immune responses require the orchestrated movement of cells in particular directions to specific locations. Cells often migrate in response to, and toward, specific external signals, which is a process called chemotaxis. In the initial phase after nerve transection, the cells involved in immune and inflammation responses, such as leukocytes, lymphocytes, mononuclear cells, and macrophages, will migrate to the injury site. In this study, we observed that the expression of genes involved in the cell migration and its positive regulation was rapidly increased before 9 h post nerve transection, and then slowly decreased. In contrast, the expression of genes involved in negative regulation of cell migration was upregulated until 7 d post nerve transection (Figure 2D). At the early phase post nerve transection, the genes involved in cell migration were mainly associated with both inflammation and immune responses. At later time points, the differentially expressed genes, such as Cx3cr1, Lbp, Cxcr4, Ifng, Ccl5, Vax1, Ednrb, and Gfra3, mainly involved migration of neurons and Schwann cells, thus playing a role in debris clearance, axonal growth and guidance, and remyelination by Schwann cells (Figure 3, Table S3.4).

#### 6) Cell proliferation

In the early stages post nerve transection, the cells involved in the immune and inflammation responses not only migrate to the injury site, but also proliferate to maintain the immune and inflammatory responses. In this study, we observe that the expression of genes involved in cell proliferation was increased sharply, and the expression of genes involved in negative and positive regulation of cell proliferation was coordinately increased to maintain a balance. Subsequently, the expression of genes involved in cell proliferation reached a high plateau during 1 d–7 d post nerve transection probably due to dedifferentiation and proliferation of Schwann cells (Figure 2E). The expression of genes with promoting functions on cell proliferation, such as IL6, IL1β, Areg, Gdnf, Ucn2, Shh, and Eger, was upregulated, while the expression of genes with inhibiting functions on cell proliferation, such as Cyp1a1, was downregulated (Figure 3, Table S3.5).

At the early stage post nerve transection, inflammatory and immune cells move toward the injury site under the guidance of chemokines, and then they proliferate to main inflammatory and immune responses. Therefore, the inflammatory response, immune response, cell migration and cell proliferation involved many identical differentially expressed genes (Table S3.2−S3.5). The expression of genes involved in inflammatory response and cell migration reached the maximum at 9 h post transection. At the later time points, however, the expression of genes involved in inflammation was decreased gradually, whereas that in immune response was increased to clear some cell debris and byproducts of inflammation response. In addition, Schwann cells need dedifferentiate and proliferation to form a Bünger's band, swallow debris, and create a microenvironment for PNS regeneration, and thus cell proliferation process maintain high level at 1–7 d post transection.

#### 7) Cell death

In this study, we observed that the expression of genes involved in the process of cell death and negative and positive regulation of cell death was promptly increased post nerve transection. In particular, the expression of genes involved in the negative regulation was increased the most rapidly, and reached the maximum at 6 h post nerve transection, and continued to be increased at 1 d post nerve transection, followed by ensuing decrease. In contrast, the expression of genes involved in positive regulation was upregulated more slowly than that involved in negative regulation, and reached to the maximum at 7 d post nerve transection. In addition, the expression of genes involved in cell death was upregulated and reached the maximum at 1 d–7 d post nerve transection (Figure 2F).

Apoptosis of adult Schwann cells is not a common phenomenon at the early stage of peripheral nerve degeneration [28]. Signal transduction through anti-apoptotic molecules is activated to prevent proximal nerves from destruction of inflammatory milieu [29]. In this study, the results indicated that negative regulation of cell death own a more significant increase than positive regulation of cell death at the early stage post nerve transection. But with time passage, a large number of inflammation and immune cells migrate to the injury site and proliferate to maintain inflammatory and immune responses after sciatic nerve injury. However, the cell number needs to maintain a relative stable value to avoid immune overreaction by establishing a relative balance of proliferation and death. Our results showed that cell death, just like cell proliferation, remained a high level at 1–7 d post nerve transaction (Figure 2E, 2F).

#### 8) Axonal regeneration and guidance

In this study, we observed that the expression of genes involved in axonal regeneration was downregulated at the first 2 time points, and the expression of genes involved in axonal guidance was upregulated at the first 2 time points and then downregulated at 1–3 h post nerve transection. Subsequently, the expression of genes involved in axonal regeneration and guidance was synchronously upregulated, reaching the peak at 7 d post nerve transection (Figure 2G). The expression of some genes involved in axonal regeneration, such as Nrep, Nefl, Apoe, was upregulated at 4 d–7 d post nerve transection (Figure 3, Table S3.7).

The growth cone, a specialized motile exploring apparatus, is located at the tip of the regenerating axon. It is guided toward its targets during nerve regeneration by the combined actions of attractive and repulsive guidance cues. In this study, following sciatic nerve transaction, an axon was found to have only a small growth, but trigger a signal for axon guidance and regeneration. Therefore, differentially expressed genes are not very numerous, especially those genes involved in axon regeneration. But the genes involved in axon guidance and regeneration exhibited the similar change trend with the same time point (7 d post transaction) when the maximum expression appeared, implying that axon guidance and axon regeneration are dependent on each other. Since axons in proximal segments underwent traumatic degeneration within the zone of injury shortly after nerve transaction [6], we assumed that the initial change of axonal regeneration and guidance might be related to a traumatic degeneration.

#### 9) Myelination

Myelin is essential for saltatory conduction of nerve signals in PNS. Following peripheral nerve injury, axonal damage and demyelination is seen [30], and demyelination enhances the ability of nerve regeneration [31]. In this study, we observed that the expression of genes involved in myelination was slowly downregulated with fluctuations at the early time points, reaching minimum at 7 d post nerve transaction, followed by an ensuing slight increase (Figure 2H). Likewise, the expression of genes involved in myelination, such as Ntf3, Mpdz, Nab1, Plp, Mal, Aspa and Mbp [32]–[34], was all down-regulated, but the expression of a negative regulator of myelination, Pou3f1 [35], was upregulated (Figure 3, Table S3.8).

Schwann cells experience a process of demyelination, dedifferentiation and proliferation after peripheral nerve injury, and after approximately 1 week, the regenerated axons are re-ensheathed and remyelinated by Schwann cells in the lesioned nerve [36]. Although axon regeneration and myelination were not very significant, the gene expression of myelination still exhibited the change trend similar to the morphological character.

### Conclusions

We applied a DNA microarray to investigate temporal expression patterns of the huge number of genes during peripheral nerve injury and regeneration, and identified the change trend of differentially expressed genes. We further used GO analysis to determine how the differentially expressed genes regulated different biological processes during peripheral nerve injury and regeneration. In vivo, most genes are in a complex and dynamic networks, and their functions are dependent on their expression abundance and microenvironment. Therefore, knowledge of the dynamic change of differentially expressed genes and related biological processes would provide a deep insight into the role of genes with spatial and temporal specificity. Following sciatic nerve transection, the 9 classes of biological processes we focused on would be activated or repressed. In this study, we analyzed the change trends of the 9 classes of biological processes and presented the expression profiles of related differentially expressed genes. Combined with morphological observation during peripheral nerve injury and regeneration, our data might help to understand the unique roles of the differentially expressed genes, especially some key regulatory genes, even to look for therapy targets for treating peripheral nerve injury. Hopefully, this study may provide a useful platform for deeply studying peripheral nerve injury and regeneration from a molecular-level perspective.

## Materials and Methods

### Animal Surgery

Adult male Sprague-Dawley (SD) rats (180–220 g, supplied by the Experimental Animal Center of Nantong University) were randomly divided into ten groups of six rats each. Each animal was anaesthetized by an intraperitoneal injection of complex narcotics (85 mg/kg trichloroac etaldehyde monohydrate, 42 mg/kg magnesium sulfate, 17 mg/kg sodium pentobarbital), and the sciatic nerve was identified and lifted through an incision on the lateral aspect of the mid-thigh of the left hind limb. A 1 cm long segment of sciatic nerve was then resected at the site just proximal to the division of tibial and common peroneal nerves, and the incision sites were then closed. To minimize discomfort and possible painful mechanical stimulation, the rats were housed in temperature- and humidity-controlled large cages with sawdust bedding after surgery. Animals were allowed free access to water and food. Animal experimentation was carried out in accordance with the NIH Guidelines for the care and use of laboratory animals (http://oacu.od.nih.gov/regs/index.htm) and ethically approved by the Administration Committee of Experimental Animals, Jiangsu Province, China (Approval ID: SYXK(SU)2007–0021).

### Tissue Collection and Microarray Experiments

Proximal sciatic nerve tissues (0.5 cm) were collected at 0.5, 1, 3, 6, and 9 h, and 1, 4, 7, and 14 d post nerve transection, respectively. Total RNA was extracted using Trizol (Life technologies, Carlsbad, CA) according to the manufacturer’s instructions. RNA quality of each sample was determined using an Agilent Bioanalyzer 2100 (Agilent technologies, Santa Clara, CA). Qualified total RNA was purified using RNeasy Mini Kit (QIAGEN, GmBH, Germany). Total RNA was amplified and labeled using a Low Input Quick Amp Labeling Kit (Agilent technologies). Labeled cRNA were further purified by RNeasy Mini kit. The quality of the labeled cRNA was again verified, and the absolute concentration was measured using a Nanodrop ND1000 spectrophotometer (NanoDrop Technologies, Wilmington, DE). The cRNA was hybridized using a Gene Expression Hybridization Kit (Agilent Technologies). Hybridization was performed at 60°C for 17 h in Hybridization Oven. The arrays were washed using a Gene Expression Wash Buffer Kit (Agilent Technologies) before stabilization and dehydration was performed (Agilent Technologies). The arrays were scanned by Agilent Microarray Scanner (Agilent Technologies) and the subsequent data compiled with Agilent feature extraction software. All steps from RNA amplification to the final scanner output were conducted by National Engineering Center for Biochip at Shanghai (China), and three biological replicates were performed for each time point.

### Data Analysis

Array normalizations and error detection were performed using Silicon Genetics’ GeneSpring GX Version 10.0 (Agilent Technologies) via the enhanced Agilent feature extraction import preprocessor. All data is MIAME compliant and the raw data has been deposited in a MIAME compliant database (NCBI Accession number: Series GSE33175, GSE30165), as detailed on the website (http://www.ncbi.nlm.nih.gov/geo/query/acc.cgi?acc=GSE33175; http://www.ncbi.nlm.nih.gov/geo/query/acc.cgi?acc=GSE30165). Values of poor quality intensities and low dependability were removed according to a “filter on flags” feature, where standardized software algorithms determined which spots were “present”, “marginal”, or “absent”. Filters were set to retain only the present and marginal values for further analysis. Data were normalized using algorithms supplied with the feature extraction software. After data normalization, independent sample T tests were performed to identify significant differences between transection and control groups (0 h). A p value of less than 0.05 and a mean expression change higher than 2 fold was considered statistically significant for further analysis. Significantly different genes were analyzed using the DAVID online analysis tool (http://david.abcc.ncifcrf.gov/) [37]. The Gene Ontology (GO) biological processes, including detection of stimulus, response to stimulus, inflammatory response, immune response, cell migration, cell proliferation, cell death, axon regeneration, axon guidance and myelination, were selected and the differentially expressed genes involved in these biological processes were screened out. The average expression profile, i.e., the centroid, was calculated according to reference [38], [39]. Key regulatory genes were z-score normalized and z-score normalized expression levels are shown in a heat map.

### Real-time Quantitative RT-PCR and Semi-quantitative RT-PCR

The same RNA samples as those described in the RNA isolation, and microarray were used to perform real-time quantitative RT-PCR analysis (qRT-PCR). Individual samples were reverse transcribed to cDNA using the Prime-Script reagent Kit (TaKaRa, Dalian, China) according to manufacturers' instructions. Real-time PCR was performed using SYBR Green Premix Ex Taq (TaKaRa) on an Applied Biosystems Stepone real-time PCR System. All reactions were run in triplicate. The relative expression of each mRNA was calculated using the comparative 2^−▵▵Ct^ method and was normalized against GAPDH. All data were expressed as mean ± SD. Pearson's correlation analysis was used to correlate qRT-PCR and microarray data. Schwann cells, fibroblasts, and L4–6 DRG neurons were harvested from sciatic nerve samples as described previously [40], [41]. The sciatic nerve and cell samples were subjected to semi-quantitative RT-PCR for determining the expression of olfactory receptors. In brief, the total RNA extraction and reverse transcription of cDNA was performed with the same protocols as the above mentioned, and thermocycler program was as follows: 5 min at 94°C; different cycles of 30 s at 94°C, 45 s at 58°C, and 30 s at 72°C (23, 30, and 35 cycles for GAPDH, olfactory receptors in sciatic nerve and Schwann cells samples, and olfactory receptors in fibroblasts and L4–6 DRG neurons samples, respectively); and 5 min at 72°C. All primers were listed in Table S5.

## Supporting Information

Figure S1 **Hierarchical clustering of key regulatory genes involved in regulation of designated biological processes during sciatic nerve regeneration.**
(TIF)

Table S1 **The expression of differentially expressed genes involved in 9 classes of biological processes at different time points post nerve transection.**
(XLS)

Table S2 **The expression of key regulatory genes in hierarchical clustering at different time points post nerve transection.**
(XLS)

Table S3 **The fold change of key regulatory genes at different time points post nerve transaction.**
(XLS)

Table S4 **The classification of key regulatory genes in terms of their expression profiles.**
(XLS)

Table S5 **Primers for RT-PCR.**
(XLS)
