# Supplementary figures and images for: Differential Gene Expression Profiling and Biological Process Analysis in Proximal Nerve Segments after Sciatic Nerve Transection
Source: PLoS One. 2013 Feb 21;8(2):e57000. doi: 10.1371/journal.pone.0057000 (PMC3578805; doi:10.1371/journal.pone.0057000)

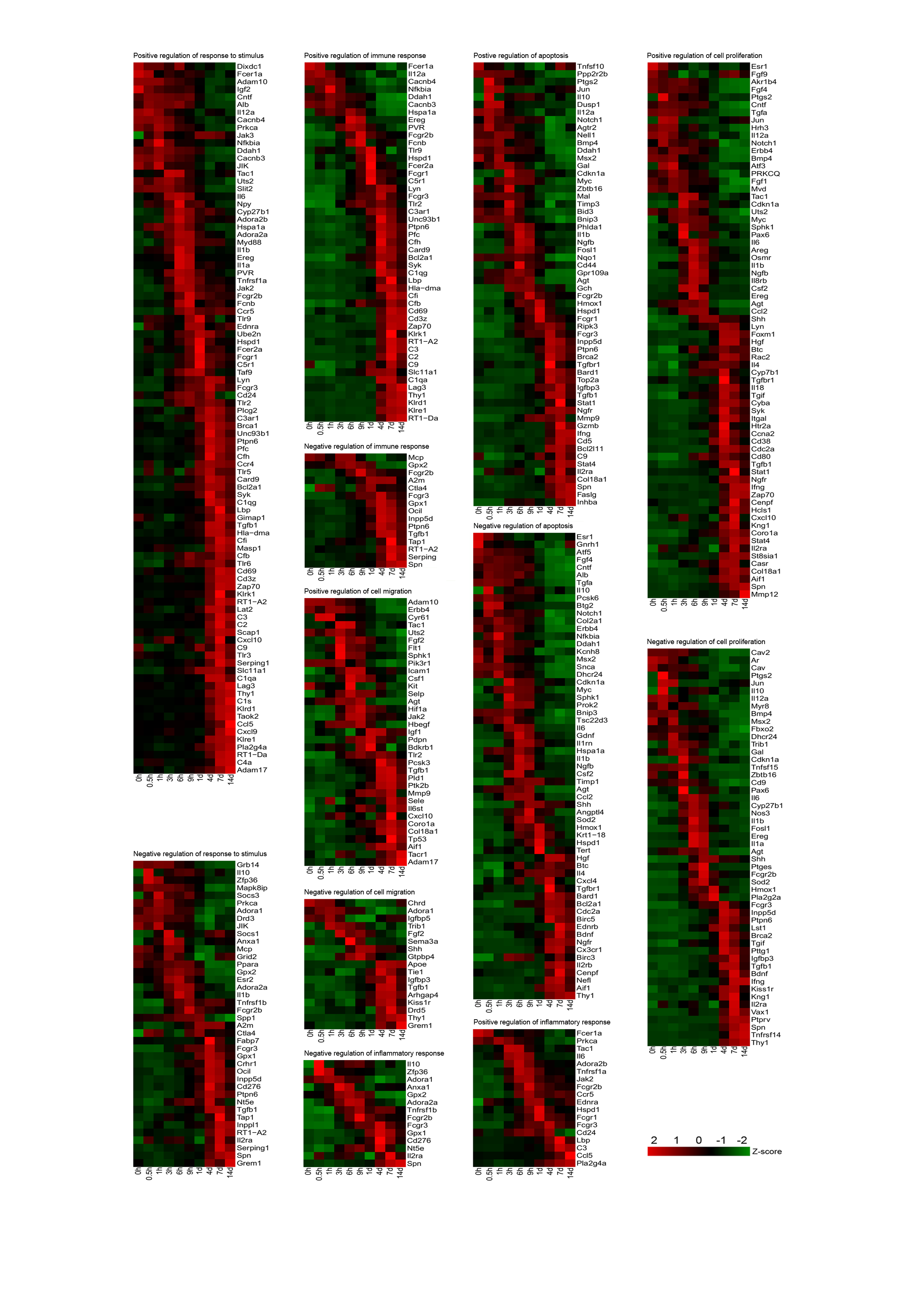

Supplement: Figure S1 — Hierarchical clustering of key regulatory genes involved in regulation of designated biological processes during sciatic nerve regeneration. (TIF) [file pone.0057000.s001.tif]
